# Screening the Efficacy and Safety of Molluscicides from Three Leaf Extracts of *Chimonanthus* against the Invasive Apple Snail, *Pomacea canaliculata*

**DOI:** 10.3390/molecules29112487

**Published:** 2024-05-24

**Authors:** Deying He, Cheng Li, Qitao Su, Yiying Lin, Zhengrong Zou

**Affiliations:** 1 College of Life Science, Jiangxi Normal University, Nanchang 330022, China; hedeey@163.com (D.H.); l1633130028@outlook.com (C.L.); 18370661917@163.com (Q.S.); 15767582760@163.com (Y.L.); 2 Key Laboratory of Protection and Utilization of Subtropic Plant Resources of Jiangxi Province, Nanchang 330022, China

**Keywords:** *Chimonanthus*, *Pomacea canaliculata*, molluscicidal activity, biochemical analysis, histopathological alterations, toxicity

## Abstract

*Pomacea canaliculata*, the invasive snail, is a host of the parasitic nematode *Angiostrongylus cantonensis*, which has adverse effects on the agriculture system and human health. This work evaluated the molluscicidal activity of petroleum ether extracts (PEEs) from three species of *Chimonanthus* against the snail *P. canaliculate*. Pcp (PEE of *C. praecox*) showed the most effective molluscicide activity. Sixty-one compounds were identified by GC-MS and the main components were terpenoids and fatty acids. The half-lethal concentration (LC_50_) of Pcp at 24 h (0.27 mg/mL) and 48 h (0.19 mg/mL) was used to evaluate the biochemical alterations in snail tissue. These sublethal concentrations caused the levels of alkaline phosphatase (ALP), alanine aminotransferase (ALT), and aspartate aminotransferase (AST) activity to increase, while acetylcholinesterase (AChE) activity decreased. Also, under LC_50_ treatment, several histological changes were observed in the hepatopancreas and foot of the snail compared with the control group. Moreover, the toxic test in rice demonstrated that Pcp has low toxicity. These results suggest that Pcp could be developed as an effective molluscicide for *P. canaliculata* control.

## 1. Introduction

*Pomacea canaliculata* (Lamarck 1822), commonly known as the golden apple snail, is native to the freshwater wetlands of South America [1,2]. It was introduced into Asian countries to manage aquatic economic organisms in the 1980s. However, it was abandoned because of the poor market response, and the snail rapidly spread to rice paddies, wild rice, and other freshwater ecosystems. The apple snail has become a serious pest for wetland plants and has a negative influence on biodiversity and the functioning of natural wetland ecosystems [3,4,5]. Hence, the species is listed as one of the world’s 100 worst invasive alien species by the International Union of Conservation of Nature, as well as being listed by the State Environmental Protection Administration of China as a significant and dangerous agricultural invasive alien organism [6,7]. Additionally, *P. canaliculata* is also the host of the parasitic nematode *Angiostrongylus cantonensis*, which can lead to eosinophilic meningitis in humans. With rapid reproduction, the risk of disease in humans will increase [8,9].

A variety of measures have been implemented to control *P. canaliculate*; the most prevalent has been the application of chemical molluscicides such as metaldehyde and niclosamide, which are synthetic. Nevertheless, these molluscicides may contaminate the ecosystem and be toxic to other nontarget aquatic organisms [10,11]. Therefore, eco-friendly and safe molluscicides are needed. Botanical pesticides have long been investigated as potent substitutes for synthetic chemical compounds due to their low toxicity to other nontarget organisms and biodegradability [12,13,14]. *Chimonanthus* is a unique genus in China that belongs to the Calycanthaceae family. According to the Flora of China, the genus contains six species: *C. praecox*, *C. nitens*, *C. salicifolius*, *C. campanulatus*, *C. zhejiangensis*, and *C. grammatus* [15,16]. Many studies have focused on *C. praecox*, *C. salicifolius*, and *C. zhejiangensis*, which have been confirmed to have abundant pharmacological activity. It was discovered that the flower extracts of these three plants exhibited antioxidant activity, and *C. praecox* showed the most potent antioxidant activity [17]. Additionally, the leaf extracts of *C. salicifolius* and *C. zhejiangensis* are named Shiliang tea, which strengthens the spleen, has antibacterial and anti-inflammatory properties, and stops diarrhea [18,19]. Regardless of this, research on the molluscicidal nature of the leaf extract of *Chimonanthus* is limited.

In this study, the chemical compositions of *C. praecox*, *C. salicifolius*, and *C. zhejiangensis* were identified and compared. Then, the molluscicidal activities of the three petroleum ether extracts were evaluated against *P. canaliculata* snails in order to screen for the most effective molluscicides. To understand the possible molluscicidal mechanisms, the histopathological and biochemical impacts of half-lethal concentration (LC_50_) were observed. We also assessed the extracts’ safety for rice plant growth in addition to evaluating the feasibility of using extracts to prevent and control snails.

## 2. Results

### 2.1. PEE Components from Three Chimonanthus Species

Sixty-one PEE compounds were identified by GC-MS from the three *Chimonanthus* species (Pcz, Pcs, and Pcp). These are shown in Table 1 and are mainly composed of terpenoids and fatty acids. Of these, 77.05% were terpenoids, including monoterpenes and sesquiterpenes, which are volatile constituents widely discovered in plant essential oils and diterpenes. Although terpenoids were the most abundant group, fatty acids were also common, especially in Pcp, and the top three compounds were ethyl linolenate, ethyl 9,12-octadecadienoic acid, and ethyl hexadecanoate.

**Figure 1:**
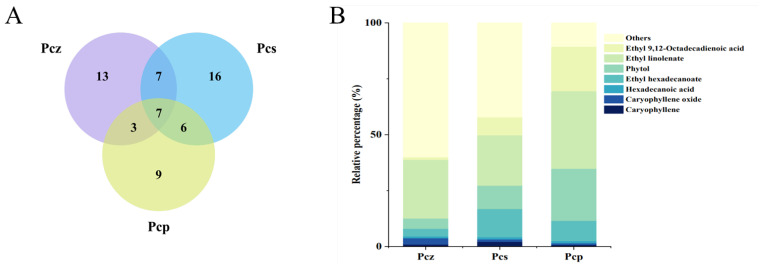
Distribution of common and unique components. Amounts of PEE chemical compounds illustrated by Venn diagram (**A**). Percentage stacked plot (**B**) of common compounds from three samples.

A Venn diagram (Figure 1A) displaying the overlapping compounds is used to visualize the relationships between the components in the three datasets. There were seven compounds that were shared by the three PEEs. These were β-caryophyllene, caryophyllene oxide, hexadecanoic acid, ethyl hexadecanoate, phytol, ethyl linolenate, and ethyl 9,12-octadecadienoic acid (Figure 1B). These compounds were distributed in different proportions in all PEEs and decreased in the following order: Pcp > Pcs > Pcz. Based on the plant material and component analysis results, natural environment and artificial cultivation play important roles in PEE components. It was found that the differences in chemical composition among the three groups were noticeable at the genus level, and it can be seen that Pcz has 13 unique ingredients, Pcs has 17, and Pcp has 9.

### 2.2. Molluscicidal Activity of PEEs

**Figure 2:**
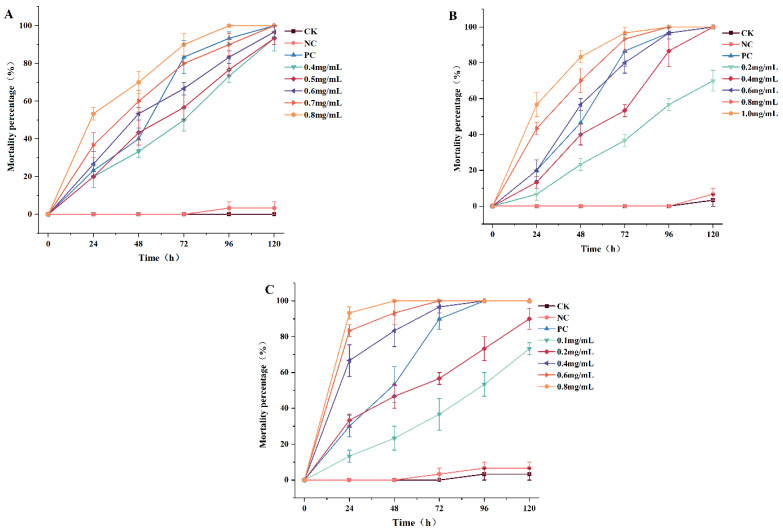
Molluscicidal activity of Pcs (**A**), Pcz (**B**), and Pcp (**C**) against *P. canaliculata*. Significance between the mean of each group is compared at the *p* < 0.05 level (n = 3).

The dose–mortality values of the three PEEs against *P. canaliculata* after various exposure intervals are presented in Figure 2. In this experiment, there were no apparently dead snails in the blank control and negative control groups after 48 h of treatment. The test demonstrated that the mortality was directly proportional to the exposure time and dose. Mortality in the positive control group exceeded 50% at 48 h and reached as high as 90% at 72 h. In the present study, Pcp showed 100% mortality at the dose of 0.8 mg/L after 48 h of exposure. The corresponding percentage mortality rates of Pcs and Pcz were 60 and 70% after the same exposure times. The 48 h LC_50_ values of Pcs, Pcz, and Pcp in *P. canaliculata* were 0.56, 0.46, and 0.19 mg/mL. In general, the results indicated that Pcp was the most effective against snails. The 24 h LC_50_ and LC_90_ values were 0.27 and 0.76 mg/mL, respectively.

### 2.3. Histopathological Studies of the Foot

**Figure 3:**
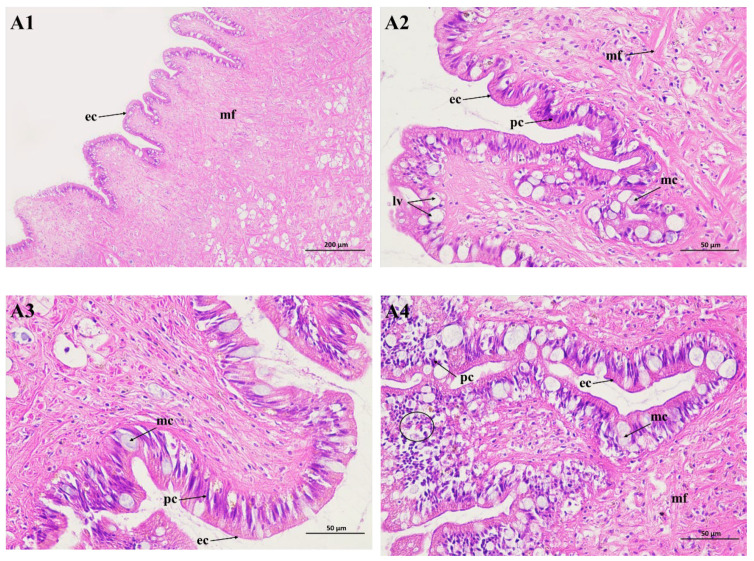
Light micrograph of the foot of *P. canaliculata* stained with hematoxylin and eosin staining (HE). **A1** (×100) and **A2** (×400) are the blank control; **A3** (×100) is the negative control; **A4** (×100) is the treated group. ec: epithelium cell; pc: pigment cell; mf: muscle fiber; lv: lipid vacuole.

The movement of snails is dependent on columnar muscle fibers generated by the conjunction of smooth muscle cells. But foot muscles in poisoned snails contract slowly. The mucous secreted from the mucous cells in the suprapedal gland enables the foot to attach to the ground. In our study, it was found that the foot cells of the snails contained epithelium cells, pigment cells, and lipid vacuoles (Figure 3(A1,A2)). In the experiment, no pathological alterations were significantly observed for the foot tissues of the negative control group. The number of lipid vacuoles decreased (Figure 3(A3)), whereas lesions in the foot tissue were observed during Pcp exposure. The number of pigment and mucous cells increased, and desquamation started in the epithelial layer. This was also seen with undifferentiated epithelial cells, while the column muscle fiber and connective tissue layer were disordered, and many empty spaces appeared within them. The results show that the feet of the snails were potently inhibited by Pcp treatment (Figure 3(A4)).

### 2.4. Histopathological Study of the Hepatopancreas

**Figure 4:**
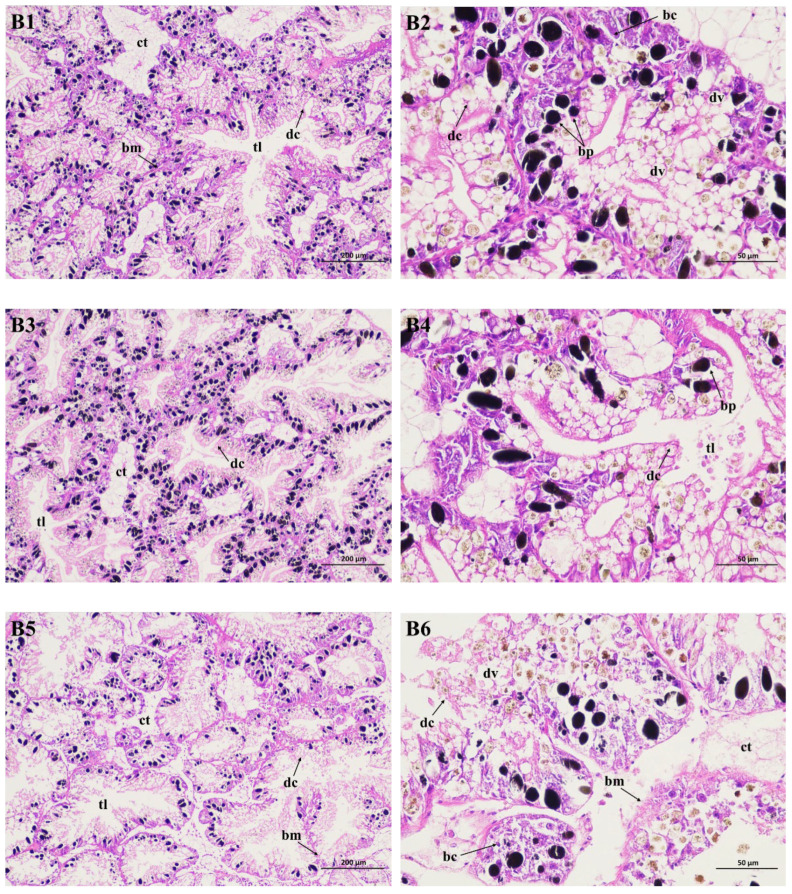
Light micrograph of the hepatopancreas of *P. canaliculata* stained with hematoxylin and eosin staining (HE). **B1** (×100) and **B2** (×400) are the blank control; **B3** (×100) and **B4** (×400) are the negative control; **B5** (×100) and **B6** (×400) are the treated group. bc: basophilic cell; dc: digestive cell; dv: digestive vacuole; bp: black particle; bm: basal membrane; ct: connective tissue; tl: tubule lumen.

The hepatopancreas is the largest gland of the gastropod digestive system, which regulates metabolism as well as the buildup and biotransformation of xenobiotics and chemicals. Using light microscopy, the blank control is shown in Figure 4(B1,B2), and is made up of digestive tubules. These tubules connect with a very thin layer of connective tissue and typically consist of two cell types: digestive cells and basophilic cells. Each tubule features a long and narrow lumen and an intermediate area interspersed with a few black particles. Digestive cells are shaped in a columnar or club pattern, and are characterized by endocytic activity and intracellular digestion. Additionally, they also contain a large number of digestive vacuoles. Basophilic cells, also known as crypt cells, are located in the crypts and are considered to be in charge of secreting enzymes for extracellular digestion. However, the hepatopancreas of the negative control (DMSO, 1% *v*/*v*) had limited alterations, and the digestive tubules appeared similarly to the CK group. Still, a small enlargement in the tubule lumen occurred (Figure 4(B3,B4)). On the other hand, the Pcp group significantly damaged the snail hepatopancreas (Figure 4(B5,B6)). After 48 h of exposure, it was found that the digestive tubules were destroyed and the tubule lumen showed a marked increase in width, with some filled with liquid materials. The digestive cells ruptured and degenerated; it was difficult to observe complete digestive vacuoles. Furthermore, the binding connective tissue lost its shape and function, and the basal membrane was thinner than in the controls.

**Figure 5:**
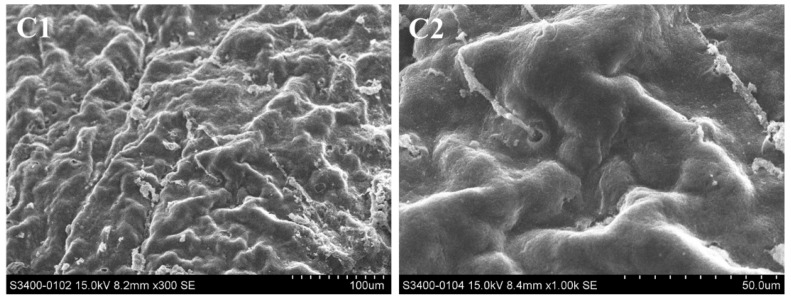

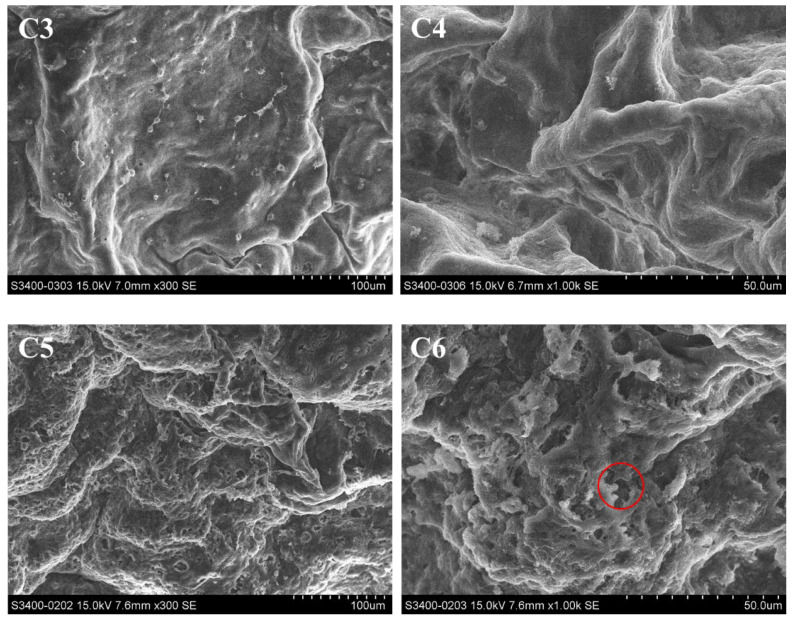
Hepatopancreas surface of *P. canaliculate,* observed by scanning electron microscopy. **C1** (×300) and **C2** (×1000) are the blank control; **C3** (×300) and **C4** (×1000) are the negative control; **C5** (×300) and **C6** (×1000) are the treated group.

The surface structure of the hepatopancreas was observed by scanning electron microscopy (SEM), and changes were recorded (Figure 5). In the control groups, there were folds and circular pores on the surface of the hepatopancreas and the pores were surrounded by fine filaments of connective tissue (Figure 5(C1–C4)). After exposure to Pcp (0.27 mg/mL) for 48 h, the hepatopancreas surface had erosive damage and the fine filaments of connective tissue disappeared. As the red circle shows, clear evidence of damage to the surface was visible.

### 2.5. Biochemical Analysis

**Figure 6:**
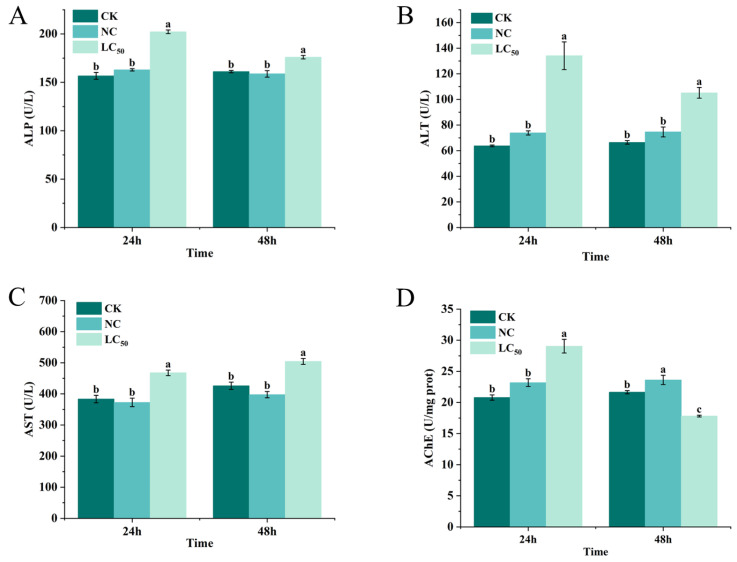
The enzyme activities in *P. canaliculata* snails exposed to LC_50_ of Pcp after 24 and 48 h. Values represent the mean ± SE (n = 3). ALP activity (**A**); ALT activity (**B**); AST activity (**C**); AChE activity (**D**). Bars marked with different letters indicate a significant difference based on the Waller–Duncan test at *p* < 0.05.

The effects of Pcp treatment with the sublethal doses of 0.27 and 0.19 mg/mL (LC_50_) on the biochemical parameters of the snails were investigated (Figure 6). ALP activity in the snail tissues increased after 24 h and 48 h of Pcp treatment with LC_50_, in comparison to the blank control, while the negative control had a less marked increase (Figure 6A). As shown in Figure 6B, the snails exposed to Pcp treatment exhibited a considerable augmentation in ALT activity after 24 h and 48 h. Moreover, the increase was greater at 24 h than at 48 h. Likewise, the level of AST increased following the treatment with Pcp. The 0.27 mg/mL dose caused an increase of more than 0.19 mg/mL (Figure 6C). The LC_50_ after 24 h of treatment resulted in a notable increase in AChE activity in the snails compared to the CK group. On the contrary, the LC_50_ after 48 h of treatment caused significant inhibition, which reduced activity by 17.7% (Figure 6D).

### 2.6. Effects of Pcp Treatment on Plant Growth

**Figure 7:**
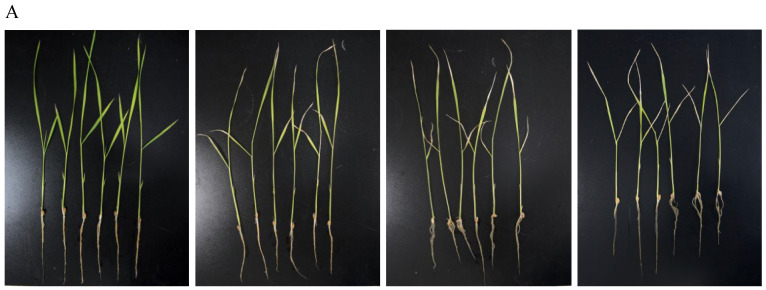

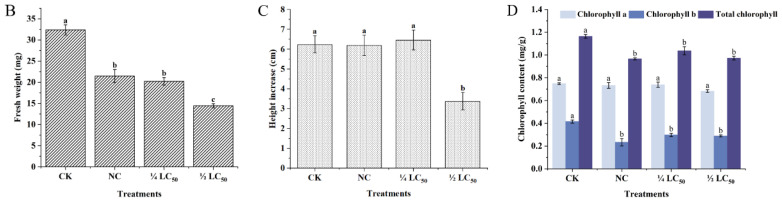
Effect of Pcp addition (**A**) on biomass production (**B**), growth in the shoots (**C**), and relative chlorophyll content in the rice leaves of rice plants (**D**) (n = 6) after 5 days of treatment. Bars marked with the same letter for each group do not differ significantly according to the Waller–Duncan test at *p* < 0.05.

As shown in Figure 7, upon Pcp treatment, the leaves of rice plants began to show wilting and yellowing symptoms. High concentrations of Pcp may cause chlorophyll breakdown and induce leaf yellowing, and fresh weight would be reduced accordingly. Total chlorophyll content decreased by 10.3% with the concentration of 135 μg/mL (50%LC_50_), and 16.4% with 67.5 μg/mL (25%LC_50_) compared to the blank control. On the other hand, there were no significant differences in the root growth of the rice plants among each group, which increased by less than 1 mm. The plant height was found to be improved with lower concentrations, but it was reduced with higher concentrations.

## 3. Discussion

The use of pesticides in agricultural systems is unavoidable: it can reduce the losses of agriculture as well as increase production and ensure quality [20,21]. Most pesticides are produced by artificial synthesis, and over 85,000 synthetic chemicals exist today [22]. These may cause pesticide contamination that results in environmental pollution and human health problems. Thus, discovering and developing natural pesticides is critical for pest control. Petroleum ether extracts are mixtures of secondary plant metabolites and perform molluscicidal activity [23]. In this study, the chemical components of PEEs from *C. praecox*, *C. salicifolius*, and *C. zhejiangensis* mainly consisted of fatty acids and terpenoids, according to our GC-MS analysis. The three most abundant fatty acids were ethyl hexadecanoate, ethyl linolenate, and ethyl 9,12-Octadecadienoic acid, which are fatty acid ethyl esters. These have a biodiesel composition and carry the advantages of biodegradability and non-toxicity [24,25]. Furthermore, the fatty acid of *C. praecox* was found to have a specific toxic effect on *P. canaliculate*. The value of the 48 h IC_50_ is 1.343 mg/mL [26]. In the present study, the fatty acid of Pcp was the most effective, with an effect of 69.37%. The molluscicidal activity of Pcp was more effective in comparison to Pcz and Pcs as well. Consequently, the molluscicidal action of Pcp may be due to its fatty acid.

Biochemical parameters are a crucial diagnostic tool in toxicological studies and are highly sensitive indicators of changes caused by xenobiotics [27]. We evaluated the sublethal toxic effects on three metabolic enzymes (ALP, ALT, and AST) and the neurotoxic enzyme (AChE) in the snails after exposure to Pcp.

ALP is crucial for gastropods as it aids in the formation of shells and other secretory activities during protein synthesis [28]. It is also used to assess hepatocyte function [29]. In our study, the sublethal doses of Pcp caused a substantial increase in the activity of ALP, which may reveal injury to the liver [30]. This increase may be due to damage to the hepatopancreas, as well as cell necrosis caused by the effect of the insecticides, which leads to the leakage of enzymes in the cells [31].

Aminotransferases are crucial enzymes that play a crucial role in the metabolism of amino acids and connect amino acids to the metabolic pathways that are involved in generating energy [32]. AST and ALT have been proposed as biomarkers of tissue injury [33]. The hepatopancreas of the snail is an organ similar to the liver that is responsible for protein recycling, carbohydrate storage, and the formation of nitrogenous excretory products [34,35]. The obtained results indicated that, with the Pcp treatment, two enzyme activities were elevated in the hepatopancreas of the snails, which was likely due to the enhanced metabolism of the amino acids and the hepatic cells being greatly damaged [36,37].

AChE physiologically removes the neurotransmitter acetylcholine from the synaptic cleft by hydrolyzing it into choline and acetic acid. Thus, inhibiting AChE can be considered a potential adverse toxicological effect [38,39]. In the present work, it was found that the LC_50_ of Pcp caused an increase in AChE activity after 24 h, but a decrease in activity after 48 h, suggesting that Pcp’s impact on the enzyme’s activity fluctuates between activation and inhibition. The inhibition of AChE activity results in an excess accumulation of acetylcholine in the cholinergic synapses at synaptic junctions and neuromuscular junctions, which can cause alterations in behavior and function. If a pesticide inhibits AChE activity by more than 20%, it is classified as a nerve poison for organisms [40]. The increase in AChE activity might indicate a compensatory response by the snail to counteract the effects of the pesticide, attempting to hydrolyze acetylcholine more rapidly to counteract the buildup caused by the inhibition of AChE [41].

On the other hand, the study results showed that a median-lethal concentration of Pcp caused histological alterations in the hepatopancreas and foot of the *P. canaliculata*. The results are similar to those reported by several authors; Ibrahim et al. [42] reported that when treated with an LC_50_ of methomyl, after 24 h, the foot of the snail *Monacha obstructa* showed a rupture of the epithelium covering the foot and the presence of a dark-brown pigment within the epithelial covering and connective tissue. The hepatopancreas exhibited breakdown of the digestive tubules as well as vacuolization, and the number of large granules increased. Karakaş and Otludil [43] found that, when exposed to 31.7 μg/L of Cd, the foot of the snail *Lymnaea stagnalis* showed an increase in mucocytes, desquamation in the epithelial cells, and atrophy in the columnar muscle cells. In the hepatopancreas, the vacuolization of the digestive cells became prominent and the connective tissue between the tubules deteriorated. Moreover, there were findings of elevated amoebocytes, the swelling of pyramidal basophilic cells, and necrotic changes.

If used as a pesticide in agricultural fields, a substance will inevitably come into contact with rice, so it is necessary to assess the toxicity of Pcp to rice. We conducted a study to evaluate the effect of Pcp on the growth of rice plants. The results showed that at a low dose of Pcp, there was no noticeable effect on the growth of rice plants, which provides the basis for its application as a molluscicide. However, further research is needed to clarify effectiveness before it is used in the agricultural system.

## 4. Materials and Methods

### 4.1. Pomacea Canaliculata

The snails were collected from Xingguo County, Ganzhou City, Jiangxi Province, China (26.35° N, 115.39° E) in April 2023 and transferred to the laboratory in a foam box. Then, they were maintained in plastic boxes (35 × 25 × 20 cm) for 10 days with a certain level of fresh water (water renewed once a day). The boxes were covered with gauze and secured with a rubber warp to prevent the snails from escaping. The snails fed on fresh lettuce for domestication, and dead snails were cleared every day. Healthy adult snails weighing 8.5 ± 0.5 g with a shell diameter of 32 ± 3 mm were selected for the experiment.

### 4.2. Plant Extracts

The leaves of *C. salicifolius* and *C. zhejiangensis* were purchased from two local farmers in Lishui City, Zhejiang Province, China (28.34° N, 119.48° E), in October 2022 and authenticated by Professor Xinfeng Zhang (Zhejiang Agriculture and Forestry University, Hangzhou, China). The leaves of *C. praecox* were picked from trees at the Jiangxi Normal University, Nanchang City, Jiangxi Province, China (28.67° N, 116.02° E) in December 2022 and authenticated by Professor Zhengrong Zou (Jiang Xi Normal University, Nanchang, China). The leaves of three plants were dried in the shade indoors for 7 days and voucher specimens (No. 2023001-2023003) were deposited at the College of Life Science, Jiangxi Normal University. Then, the leaves were crushed to a powder and passed through a 60-mesh sieve. The powders of the three plants (500.0 g) were soaked in 70% ethanol at a ratio of 1:10 (*w*/*v*) and subjected to ultrasonic extraction for 30 min, then left at room temperature (25 ± 2 °C) for 48 h. The extracts were filtered and evaporated to dryness under reduced pressure to obtain the ethanolic extracts (EEs). The EEs of *C. zhejiangensis*, *C. salicifolius*, and *C. praecox* were 84.6 g, 94.0 g, and 63.1 g. Then, the EEs were suspended in deionized water and extracted successively with petroleum ether four times. The upper extracts were pooled and concentrated to obtain the petroleum ether extracts (PEEs) of *C. zhejiangensis* (Pcz), *C. salicifolius* (Pcs), and *C. praecox* (Pcp). The weights of them were 30.5 g, 38.5 g, and 21.6 g.

### 4.3. Gas Chromatography–Mass Spectrometry (GC-MS) Analysis

The PEEs were analyzed by GC-MS using a Thermo Trace 1300-ISQ instrument equipped with a capillary column (HP-5MS) (30 m × 0.25 mm × 0.25 µm). The temperature of the injector was set at 230 °C and the detector was set at 280 °C. For analysis, a 10 μL sample was injected in split mode at a ratio of 1:2. The initial column temperature was set at 60 °C for 5 min, increased to 120 °C at 10 °C/min, held for 5 min, further increased to 200 °C at 10 °C/min, maintained for 5 min, and finally increased to 280 °C at the same rate. The carrier gas was helium at a flow rate of 1.0 mL/min. The mass spectrometry was performed in EI mode at 70 eV with scanning from 50 to 650 amu. The ion source and transfer line temperatures were set at 250 °C. The chemical components were identified by comparing the mass spectra and retention indices (RIs) in the NIST mass spectral library. The percentage of chemical compounds was quantified by peak area normalization.

### 4.4. Investigation of Molluscicidal Activity

The molluscicidal activity of the PEEs from three *Chimonanthus* species was evaluated using the snail-immersion bioassay method. Pcz, Pcs, and Pcp were first dissolved in dimethyl sulfoxide (DMSO, 1% *v*/*v*) and subsequently introduced into dechlorinated tap water. Healthy adult snails were selected randomly and put into glass beakers (n = 10 per beaker) where they were immersed in the solutions (300 mL). Each group reached the final concentration (Pcs: 0.4, 0.5, 0.6, 0.7, and 0.8 mg/mL; Pcz: 0.2, 0.4, 0.6, 0.8, and 1.0 mg/mL; Pcp: 0.1, 0.2, 0.4, 0.6, and 0.8 mg/mL). Three replicates were performed for each concentration, along with dechlorinated tap water serving as the blank control (CK), DMSO (1% *v*/*v*) as the negative control (NC), and nicotinanilide as the positive control (PC). Nicotinanilide was dissolved in dechlorinated tap water and then diluted to 0.8 mg/L. The mortality of the *P. canaliculata* was recorded after 24, 48, 72, 96, and 120 h in each beaker, and the dead individuals were removed. If a snail did not crawl against the glass wall, it would be examined for death. According to the WHO [44], death was detected by stimulating the snails with a needle to induce a typical retreating movement.

### 4.5. Histopathological Observation

The histopathological study was carried out on the adult snails that were treated with 0.27 mg/mL of Pcp to determine the toxic effect on the foot and hepatopancreas. Dechlorinated water served as the blank control, while DMSO (1% *v*/*v*) served as the negative control. Following 48 h of treatment, in both the experimental and control groups, the shell of the snail was broken and the soft tissues were dissected. The organ samples were stored in 4% paraformaldehyde for hematoxylin and eosin staining (HE) and observed under a microscope (Nikon ECLIPSE Ci, Nikon, Tokyo, Japan). The other portion of the hepatopancreas was stored in electron microscope fixative (2.5% glutaraldehyde) for scanning electron microscopy (HITACHI S-3400N, HITACHI, Tokyo, Japan).

### 4.6. Biochemical Analysis

The snails were subjected to the LC_50_ of Pcp and evaluated at 24 h and 48 h. The hepatopancreases of the alive snails were dissected from each group and washed with ice-cold saline (0.9% NaCl). Then, the tissues were weighted and homogenized in nine volumes (*w*/*v*) of ice-cold saline solution. In a frozen centrifuge, the homogenates were centrifuged at 3500 rpm for 15 min at 4 °C. The supernatants were frozen and used to measure the enzyme activity of ALP, ALT, AST, and AChE.

The activity of ALT and AST was determined at 510 nm using a Spectrum Diagnostic Kit purchased from Nanjing Jiancheng Bioengineering Institute, following the instructions. The activity of ALP was measured at 405 nm, using a kit purchased from Suzhou Grace Biotechnology Co., Ltd. (Suzhou, China) with p-Nitrophenyl phosphate as the substrate. For AChE activity, acetylcholine was used as the substrate, and activity was assessed as 412 nm utilizing a Grace Diagnostics kit.

### 4.7. Effect on Rice Plant Growth

The seeds of rice used in the present test were provided by a farmer from Jiangsu Province, Suzhou City, China. Seeds with complete grains were selected and disinfected with 2% hydrogen peroxide (H_2_O_2_) for 10 min. After rinsing with deionized water, they were immersed in deionized water for 5 h. Then, these seeds were germinated in several plastic boxes covered with wet filter paper, and the germinated seeds were grown in a plastic pot (10 per pot) with IRRI nutrient solution [45] for 13 days under greenhouse conditions (14:10 light/dark, 25 ± 2 °C)., Different concentrations of Pcp were applied up to the three-leaf stage: 135 μg/mL (50%LC_50_) and 67.5 μg/mL (25%LC_50_). The Pcp was dissolved in DMSO (0.5% *v*/*v*). The blank control was a nutrient solution with nothing added and the negative control was a nutrient solution added DMSO (0.5% *v*/*v*). The corresponding solution in pots was replaced every 3 days during the test period, which lasted 18 days.

The height of the rice plant was measured at the start and end of the five-day treatment exposure. The length increase in the plant root was measured with the same method. Plant samples were selected randomly, and the leaves were removed from the stem. The separated plant leaves were weighed and recorded. Then, the leaves were thoroughly ground up and the chlorophyll content was extracted using 95% ethanol. Briefly, 200 µL of extract was scanned at 649 and 665 nm wavelengths with a microplate reader (BioTek Synergy H1, Agilent, Santa Clara, CA, USA).

### 4.8. Statistical Analysis

Statistical analysis was performed with SPSS 26.0 software. The half-lethal concentration values (LC_50_), 95% confidence limits (CLs), and slopes were calculated by the Probit method. Data are described as the mean ± standard error (SE), which was conducted using one-way ANOVA, followed by the Waller–Duncan test at a significance level of *p* ≤ 0.05. The 95% confidence interval of the Fisher least significant difference (LSD) method was used to compare means between treatments.

## 5. Conclusions

This study compared the molluscicidal activity of three leaf extracts of *Chimonanthu* for the first time and revealed that Pcp was the most effective pesticide. The GC-MS analysis showed that the chemical components of the extract are terpenoids and fatty acids, and that these could serve as potential alternatives to synthetic molluscicides for controlling snails. Furthermore, to elucidate the mechanisms of toxic action, we evaluated the biochemical and histopathological alterations in the snails after exposure to the sublethal concentration of Pcp, and found that it caused metabolic disorders and organ damage. Additionally, a low dose of Pcp has a good safety profile, as it exhibited low toxicity to rice plant growth. This study has assessed Pcp as a safe molluscicide to control *P. canaliculata*.

## Figures and Tables

**Table 1:** Chemical components of PEEs from three *Chimonanthus* species.

No.	Compound	Formula	CAS Number	RI	Relative Peak Area %		
					Pcz	Pcs	Pcp
1	Eucalyptol	C_10_H_18_O	00470-82-6	1038	-	2.10	-
2	*endo*-Borneol	C_10_H_18_O	00507-70-0	1166	0.43	-	-
3	4-Terpineol	C_10_H_18_O	00562-74-3	1169	-	0.24	-
4	*α*-Terpineol	C_10_H_18_O	00098-55-5	1173	1.32	7.93	-
5	Citronellol	C_10_H_20_O	00106-22-9	1217	-	0.48	-
6	Geraniol	C_10_H_18_O	00106-24-1	1254	0.79	1.62	-
7	Linalyl acetate	C_12_H_20_O_2_	00115-95-7	1256	-	-	0.15
8	Terpinyl acetate	C_12_H_20_O_2_	00080-26-2	1343	0.76	2.42	-
9	Citronellol acetate	C_13_H_24_O_2_	00150-84-5	1354	0.36	-	-
10	α-Copaene	C_15_H_24_	03856-25-5	1376	-	0.51	0.16
11	β-Elemene	C_15_H_24_	00515-13-9	1394	-	-	0.15
12	*(E)*-*β*-Caryophyllene	C_15_H_24_	00087-44-5	1417	0.98	2.21	0.79
13	α-Humulene	C_15_H_24_	06753-98-6	1452	10.70	0.38	-
14	Germacrene D	C_15_H_24_	23986-74-5	1484	-	1.43	-
15	*(E,E)*-*α*-Farnesene	C_15_H_24_	00502-61-4	1496	-	0.41	-
16	α-Calamene	C_15_H_22_	01460-96-4	1512	0.48	-	-
17	Cubebol	C_15_H_26_O	23445-02-5	1516	-	0.30	0.34
18	Cadina-1(10),4-diene	C_15_H_24_	00483-76-1	1519	-	0.97	-
19	Butylated hydroxytoluene	C_15_H_24_O	00128-37-0	1533	-	-	0.20
20	Elemol	C_15_H_26_O	00639-99-6	1537	-	1.71	-
21	α-Copaen-11-ol	C_15_H_24_O	41370-56-3	1547	-	0.34	-
22	Hedycaryol	C_15_H_26_O	21657-90-9	1559	-	-	0.45
23	*trans*-Nerolidol	C_15_H_26_O	40716-66-3	1564	-	0.44	-
24	Spathulenol	C_15_H_24_O	06750-60-3	1566	-	0.23	-
25	Dendrolasin	C_15_H_22_O	23262-34-2	1571	1.19	-	-
26	Germacrene-4-ol	C_15_H_26_O	74841-87-5	1574	-	-	0.23
27	Caryophyllene oxide	C_15_H_24_O	01139-30-6	1578	2.85	1.08	0.73
28	Calarene epoxide	C_15_H_24_O	68926-75-0	1592	1.91	-	-
29	Isoaromadendrene epoxide	C_15_H_24_O	/	1594	1.95	-	0.10
30	Carotol	C_15_H_26_O	00465-28-1	1596	-	0.37	-
31	Cedrol	C_15_H_26_O	00077-53-2	1605	-	-	0.19
32	Epiglobulol	C_15_H_26_O	88728-58-9	1608	-	0.54	0.26
33	*γ*-Eudesmol	C_15_H_26_O	01209-71-8	1630	-	0.53	-
34	*τ*-Muurolol	C_15_H_26_O	19912-62-0	1632	0.71	1.78	-
35	Cubenol	C_15_H_26_O	21284-22-0	1643	-	-	1.88
36	*β*-Eudesmol	C_15_H_26_O	00473-15-4	1645	-	1.17	-
37	*α*-Eudesmol	C_15_H_26_O	00473-16-5	1652	-	0.76	-
38	*α*-Cadinol	C_15_H_26_O	00481-34-5	1663	0.23	-	-
39	*α*-Santalol	C_15_H_24_O	00115-71-9	1681	0.34	5.02	-
40	*(Z,Z)*-2,6-Farnesol	C_15_H_26_O	04602-84-0	1695	1.01	-	-
41	Aromadendrene epoxide II	C_15_H_24_O	85710-39-0	1706	6.97	-	0.44
42	Shyobunol	C_15_H_26_O	35727-45-8	1709	2.22	-	-
43	Longifolol	C_15_H_26_O	00469-27-2	1720	-	-	0.22
44	trans-Farnesol	C_15_H_26_O	00106-28-5	1722	3.64	2.53	-
45	*(Z)*-α-Bisabolene epoxide	C_15_H_24_O	111536-37-9	1733	1.26	-	-
46	Tetradecanoic acid	C_14_H_28_O_2_	00544-63-8	1765	-	-	0.28
47	Ethyl tetradecanoate	C_16_H_32_O_2_	00124-06-1	1793	-	0.25	0.19
48	Ledene oxide-I	C_15_H_24_O	/	1890	4.43	-	-
49	Corymbolone	C_15_H_24_O_2_	97094-19-4	1898	6.35	-	-
50	Methyl palmitoleate	C_17_H_32_O_2_	01120-25-8	1932	-	0.40	-
51	9-Hexadecenoic acid	C_16_H_30_O_2_	02091-29-4	1942	1.70	-	-
52	Hexadecanoic acid	C_16_H_32_O_2_	00057-10-3	1964	0.81	0.97	1.03
53	Ethyl 9-hexadecenoate	C_18_H_34_O_2_	54546-22-4	1978	2.75	-	0.18
54	Ethyl hexadecanoate	C_18_H_36_O_2_	00628-97-7	1993	3.41	12.63	9.04
55	Geranylgeraniol	C_20_H_34_O	24034-73-9	2201	2.12	-	-
56	Phytol	C_20_H_40_O	00150-86-7	2111	4.61	10.46	23.27
57	9,12,15-Octadecatrienoic acid	C_18_H_30_O_2_	01955-33-5	2117	-	4.37	3.10
58	9,12-Octadecadienoic acid	C_18_H_32_O_2_	00060-33-3	2144	-	0.74	0.96
59	Ethyl linolenate	C_20_H_34_O_2_	01191-41-9	2173	26.28	22.50	34.72
60	Ethyl octadecanoate	C_20_H_40_O_2_	00111-61-5	2194	-	0.77	-
61	Ethyl 9,12-Octadecadienoic acid	C_20_H_36_O_2_	07619-08-1	2515	1.85	8.08	19.87
Total (%)					94.41	93.65	98.93
Oxygenated monoterpenes (%)					3.33	14.79	0.15
Sesquiterpene hydrocarbons (%)					12.16	5.91	1.10
Oxygenated sesquiterpenes (%)					35.06	16.80	5.04
Oxygenated diterpene (%)					6.73	10.46	23.27
Fatty acid compounds (%)					37.13	50.71	69.37

## Data Availability

The data presented in this study are available on request from the corresponding authors.
